# Immunosuppression-related neurological disorders in kidney transplantation

**DOI:** 10.1007/s40620-020-00956-1

**Published:** 2021-01-22

**Authors:** Irene Faravelli, Daniele Velardo, Manuel Alfredo Podestà, Claudio Ponticelli

**Affiliations:** 1 Neuroscience Section, Department of Pathophysiology and Transplantation (DEPT), Dino Ferrari Centre, Università degli Studi di Milano, Milan, Italy; 2 Neurology Unit, Foundation IRCCS Ca’ Granda Ospedale Maggiore Policlinico, Milan, Italy; 3 Renal Division, ASST Santi Paolo e Carlo, Department of Health Sciences, Università degli Studi di Milano, Milan, Italy; 4 Milano, Italy

**Keywords:** Immunosuppressive drugs, Nervous system, Neurological disorders, Renal transplantation

## Abstract

A large number of neurological disorders can affect renal transplant recipients, potentially leading to disabling or life-threatening complications. Prevention, early diagnosis and appropriate management of these conditions are critical to avoid irreversible lesions. A pivotal role in the pathogenesis of common post-transplant neurological disorders is played by immunosuppressive therapy. The most frequently administered regimen consists of triple immunosuppression, which comprises a calcineurin inhibitor (CNI), a purine synthesis inhibitor and glucocorticoids. Some of these immunosuppressive drugs may lead to neurological signs and symptoms through direct neurotoxic effects, and all of them may be responsible for the development of tumors or opportunistic infections. In this review, after a brief summary of neurotoxic pathogenetic mechanisms encompassing recent advances in the field, we focus on the clinical presentation of more common and severe immunosuppression-related neurological complications, classifying them by characteristics of urgency and anatomic site. Our goal is to provide a general framework that addresses such clinical issues with a multidisciplinary approach, as these conditions require.

## Introduction

A wide array of neurological complications may impact on the outcome of renal transplant recipients, possibly leading to disabling or life-threatening diseases. Some of these complications are caused by the disorders that led to transplantation and by dialysis [1]. Since criteria to admit donors and recipients to a transplantation program have been considerably expanded in recent years, elderly individuals and patients with severe comorbidities may now be considered suitable candidates for kidney transplantation. On one hand, this has allowed to expand the number of recipients and donors, but on the other it has increased the risk of extra-renal complications, including those affecting the central and peripheral nervous system.

The most crucial role in the pathogenesis of post-transplant neurological disorders is played by immunosuppressive drugs. The choice of immunosuppressive regimens varies widely across transplant programs, nevertheless, the most frequently adopted schedule rests on triple immunosuppression comprising a calcineurin inhibitor (CNI) such as tacrolimus (TAC) or cyclosporine (CsA), a purine synthesis inhibitor, such as mycophenolate (MPA) or azathioprine, and glucocorticoids. Some centers have been gaining experience with inhibitors of the mammalian target of rapamycin (mTOR) and, more recently, with belatacept. Anti-thymocyte globulins (ATG), anti-CD52 (alemtuzumab), anti-CD25/IL-2R (basiliximab) and anti-CD20 (rituximab) monoclonal antibodies are also frequently used for induction therapy or treatment of rejection episodes. The proteasome-inhibitor bortezomib has also been used for the management of antibody-mediated rejection in selected cases. Some of these immunosuppressive drugs may exert direct neurotoxic effects, and all of them may be involved in the development of tumors or opportunistic infections [2].

**Fig. 1:**
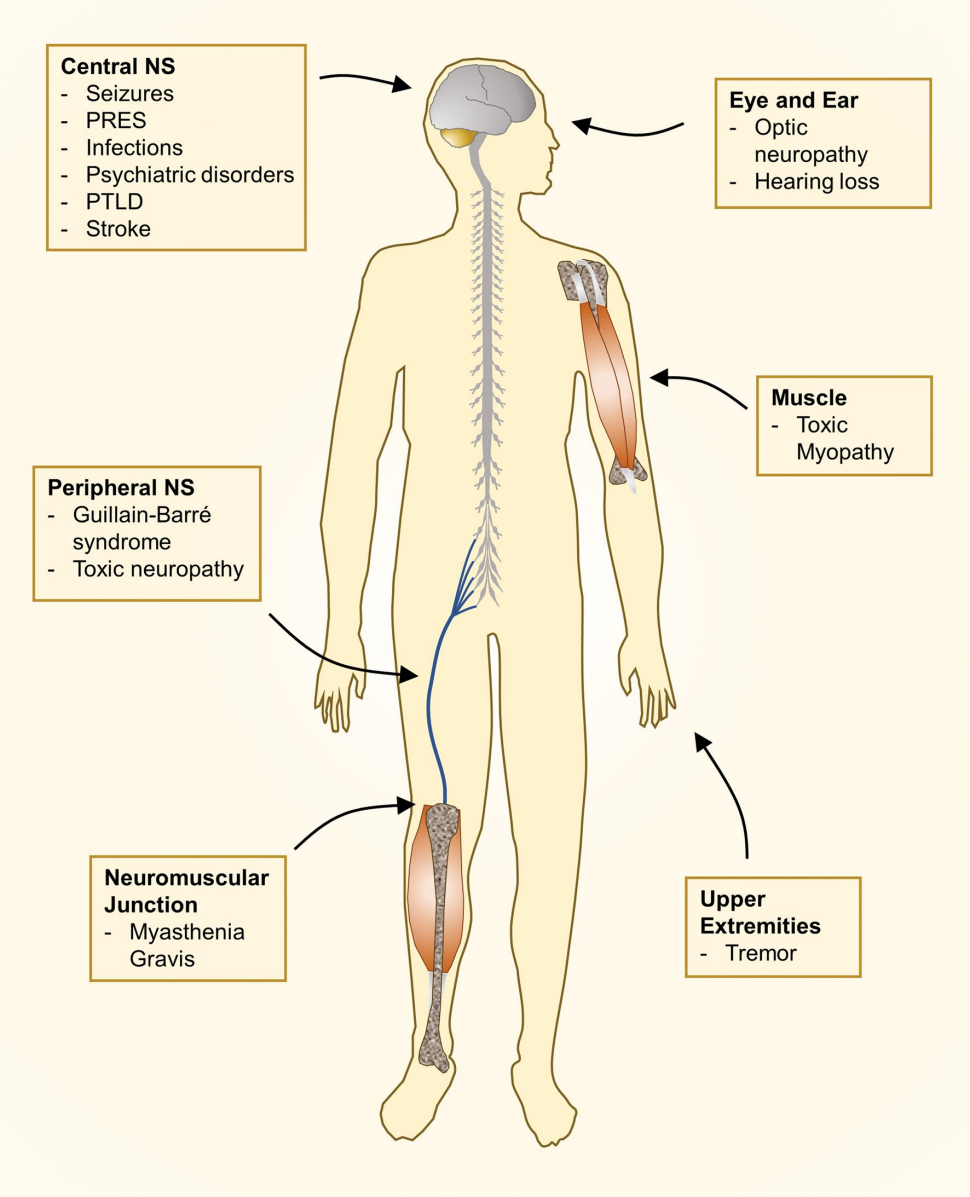
Neurological complications in renal transplant recipients by anatomic site. *NS* nervous system, *PRES* posterior reversible encephalopathy syndrome, *PTLD* post-transplant lymphoproliferative disorders

In this review, after a brief overview of neurotoxic pathogenetic mechanisms encompassing recent advances in the field, we focus on the clinical presentation of more common and severe neurological complications. This work deals with the neurological presentations most frequently caused by treatment with immunosuppressive drugs, classifying them by characteristics of urgency and anatomic site (central or peripheral nervous system) (Fig. 1).

## Direct effects of immunosuppressive drugs

### Calcineurin inhibitors

Calcineurin is a calcium- and calmodulin-binding protein phosphatase that participates in a wide array of cellular processes and calcium-dependent signal transduction pathways, including T-cell activation. CsA and TAC inhibit calcineurin to a similar degree, inducing downstream blockade of IL-2 signaling, ultimately interfering with T-cell activation, proliferation and differentiation. Calcineurin is highly expressed in the central nervous system, particularly in neurons vulnerable to ischemic and traumatic injury.

**Fig. 2:**
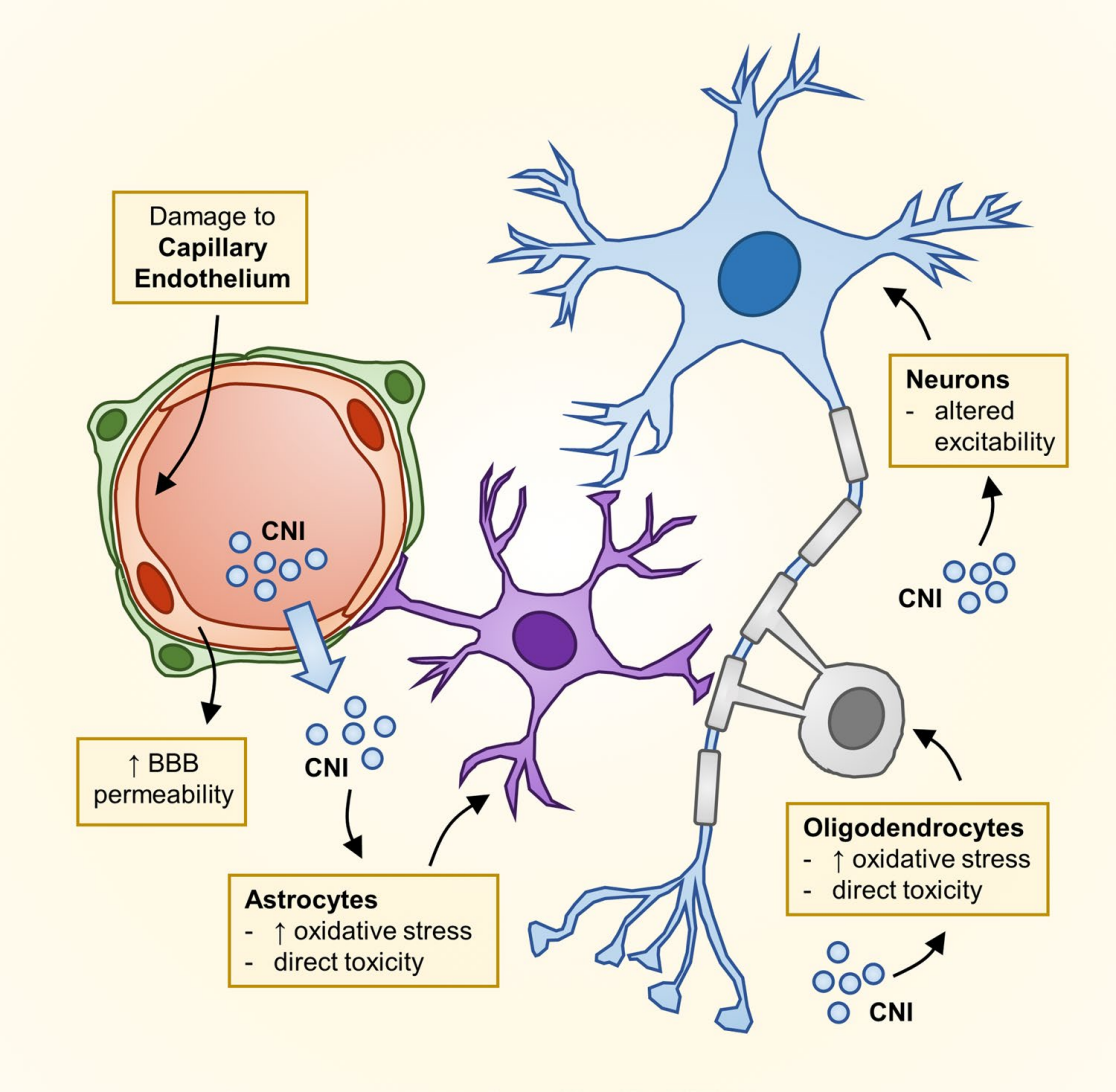
Proposed mechanisms of neural toxicity due to calcineurin inhibitors (CNIs). Blood–brain barrier (BBB) permeability can be altered by CNI-induced damage to the capillary endothelium or by other concomitant disorders (e.g. infection and inflammation). CNI crossing of the damaged BBB may lead to altered neuronal excitability and could result in direct toxicity on glial cells (astrocytes and oligodendrocytes), which are particularly susceptible to these agents due to their high calcineurin content

Several mechanisms may be involved in CNI-related toxicity on the central nervous system (CNS) (Fig. 2). CNIs do not readily cross the blood–brain barrier (BBB) in physiological states, however, these agents may induce apoptosis of brain capillary endothelial cells and inhibit P-glycoprotein (P-gp) function, thus altering BBB permeability [3, 4]. These effects are particularly relevant when other, concomitant causes of BBB permeability alteration are present (e.g. inflammation). After diffusing across the BBB, CNIs may exert selective toxic effects on glial cells [5] and oligodendrocytes [6], the latter being particularly susceptible to CNI-induced damage due to their high calcineurin content. CNIs may also directly alter mitochondrial function, increasing oxidative stress in glial cells [7]. Moreover, both CsA and TAC modulate the activity of excitatory and inhibitory neurotransmitter receptors [8, 9], leading to altered excitability properties and resulting in membrane depolarization, which have been proposed as an additional mechanism of CNI neurotoxicity [10].

In addition to their direct effects on neural cells, CNIs may lead to activation of the major vasoconstriction systems, i.e. the renin–angiotensin and endothelin systems, and increase sympathetic system activity. In addition, CNIs inhibit nitric oxide synthesis and nitric oxide-mediated vasodilation. Altogether, these processes cause vasoconstriction and endothelial dysfunction [11], which result in systemic hypertension, local ischemia and cerebral edema. Moreover, if endothelial integrity is disrupted, CNIs may damage astrocytes that provide structural, trophic and metabolic support to neurons. On the other hand, animal models have suggested that CsA may prevent brain damage from forebrain ischemia when allowed to pass the BBB, whereas TAC is significantly less effective [12–16]. This neuroprotective effect might reflect inhibition of cyclophilin D, but the exact mechanism and its clinical potential still need to be fully elucidated [17].

### Purine synthesis inhibitors

Azathioprine and mycophenolate interfere with nucleotide synthesis with different mechanisms. Azathioprine is a mercaptopurine prodrug, whose pharmacological activity rests on the formation of two active intracellular nucleotides: thioinosinic acid inhibits the de novo pathway of purine synthesis, while 6-thioguanine interferes with the purine salvage pathway. These actions result in depletion of cellular purine stores and inhibition of DNA/RNA synthesis, hampering lymphocyte proliferation. The immunosuppressive effects of azathioprine are more potent on T cells compared to B cells, owing to intrinsic metabolic differences in these cells. Mycophenolate salts are prodrugs releasing active mycophenolic acid (MPA), which reversibly inhibits inosine monophosphate dehydrogenase and results in a marked reduction of guanosine triphosphate necessary for DNA synthesis. Neurological symptoms directly induced by purine synthesis inhibitors are rare and mild, usually manifesting as depression and headaches. Cases of progressive multifocal leukoencephalopathy (PML), caused by John Cunningham Virus (JCV) infection, have been reported in renal transplant recipients or lupus patients treated with MPA, and thus the Food and Drug Administration warned against this possible risk.

### Glucocorticoids

Synthetic glucocorticoids exert a wide array of anti-inflammatory and immunosuppressive activities that are mainly mediated by their genomic effects. Glucocorticoids bind to their specific cytosolic receptors, enter the nucleus and either activate response elements that induce anti-inflammatory genes, or repress inflammatory transcription factors (e.g. NF-kB or activator protein-1). These drugs may also produce non-genomic effects with rapid onset and short duration, whose mechanism of action is incompletely understood. At least part of this activity seems to be mediated by membrane receptors that can modulate anti-inflammatory and antioxidant effects [18].

Brain cells express two types of corticosteroid receptors, i.e. mineralocorticoid and glucocorticoid receptors, which differ in distribution and affinity and can exert modulatory effects on a variety of brain functions from early development through late life. The potential effects of glucocorticoids on neuronal activity are determined predominantly by receptor distribution since glucocorticoids easily pass the BBB and virtually reach all brain cells, although local enzymatic conversion and the degree of cell accessibility contribute to the actual intracellular concentration [19].

The use of glucocorticoids in pediatric transplant recipients may be particularly problematic. Exposure to high-dose glucocorticoids in early life can significantly affect the hypothalamic–pituitary–adrenal axis and increase the susceptibility to develop metabolic, neuropsychiatric and neurodegenerative disorders [20]. In addition, elevated glucocorticoid levels and prolonged exposure to stressful conditions can induce structural remodeling of neurons with synaptic loss and maladaptive alterations in glial functions [21].

### mTOR inhibitors

Sirolimus (rapamycin) and its derivate everolimus inhibit the mTORC1 complex, a master regulator of cell growth and metabolism, interfering with signals leading to T-cell proliferation. Both mTOR inhibitors can cross the BBB, but there is considerable uncertainty regarding their potential neuroprotection or neurotoxicity. In the nervous system, the mTOR pathway regulates axonal sprouting, axonal regeneration and myelination, ion channel and receptor expression, as well as dendritic spine growth [22]. mTOR signaling can also regulate mitochondrial function [23] and enhance synaptic activity by promoting the synthesis and surface expression of AMPA receptors, which are members of the ionotropic class of glutamate receptors [24]. On the other hand, chronic activation of mTOR signaling can aggravate vascular senescence and ischemic injury, and may contribute to neuroinflammation and autophagy dysfunction in degenerative neurological diseases [25]. In addition, mTOR is involved in the upregulation of glutamate transporter 1 that is linked to several neuronal disorders such as stroke, Alzheimer’s disease, and amyotrophic lateral sclerosis [26]. Accordingly, mTOR inhibitors are not considered neurotoxic drugs per se, and may even play a neuroprotective role by suppressing the pharmacological effects of the mTOR pathway. However, some data suggest that sirolimus could enhance CsA-induced formation of reactive oxygen species and their negative metabolic effects in brain cells, while everolimus seems to antagonize CsA effects [27].

### Other immunosuppressive agents

Rituximab is a monoclonal antibody with a high affinity for the CD20 antigen, a membrane protein expressed on B cells. Reports of PML cases in patients treated with Rituximab have raised concerns about its use in renal transplant recipients, since the risk seems to be higher in patients treated with multiple immunosuppressive drugs [28]. However, the incidence of this potentially fatal complication seems to be very low both in patients with autoimmune disorders and in renal transplant recipients [29, 30]. Alemtuzumab is a humanized anti-CD52 monoclonal antibody that is used as induction or anti-rejection therapy in renal transplant recipients. Though alemtuzumab and other T cell-depleting drugs have not been conclusively demonstrated as a direct cause of neurotoxicity, they have a profound impact on the immune system, and may therefore predispose to infections and their neurological complications in the transplantation setting [31].

Rabbit ATG are polyclonal antibodies that reduce the number of circulating T cells through cell lysis. Bortezomib, a broad-spectrum proteasome inhibitor, may cause neurotoxic effects through a dysregulation of neurotrophins and blockade of nerve growth factor-mediated neuronal survival induced by NF-κB inhibition. Belatacept is a fusion protein with high affinity for CTLA-4 (CD152), a cell surface protein transiently expressed on T cells that attenuates their activation. This drug blocks T-cell costimulation and may increase the risk of post-transplant cerebral lymphoma in Epstein Barr virus- (EBV) negative patients who receive a kidney from EBV positive donors [32]. Finally, Eculizumab, a monoclonal antibody that binds to the terminal complement component C5 and blocks its activating cleavage, is currently used in renal transplant recipients diagnosed with atypical hemolytic uremic syndrome; complement inhibition can increase the risk of infection by capsulated bacteria (e.g. *S. pneumoniae*, *N. meningitidis*), which can be however effectively prevented by vaccination against these pathogens.

## Central nervous system

### Acute clinical presentations

#### Seizures

Seizures have been reported in 6–36% of transplant recipients [33] and the most frequent causes include immunosuppressive drugs. CsA is epileptogenic and responsible for seizure in 2–6% of patients, while TAC has been associated to seizures with a slightly higher frequency (5–11%) [34]. A study evaluating 132 patients who underwent kidney transplantation described seizures in 16.7% of them, none of whom had a prior history of seizures [35]. It is worth mentioning that some of these studies report higher CNI targets, which can contribute to the increased risk of seizure. However, seizures may occur even with actual standard doses of CNI, especially in pediatric patients with underlying metabolic abnormalities (hyponatremia, hypomagnesemia, hypoglycemia) and infections.

Intravenous methylprednisolone pulses (MPP), 500–1000 mg per dose, are the standard treatment for acute rejection and are frequently used for induction therapy. Although MPP therapy may be considered for possible treatment in patients with focal epilepsy, exceptional cases of seizures have been reported after intravenous administration of steroids [36]. Complications occurred in severely affected patients and when treatment was injected rapidly and through a central venous line. To minimize the neurological side-effects of intravenous high-dose MPP, they should be infused in a peripheral vein over 30–60 min and outpatients should be monitored for at least 2 h after infusion [37].

#### Stroke

Several studies have consistently reported a higher frequency of cerebral vascular events in kidney transplant recipients compared to the general population, with an incidence of 5% in the first year and 9.4% in the second year post-surgery [37–39]. This is in part due to predisposing factors such as vasculopathy, accelerated atherosclerosis, hypertension and diabetes, which are among the most frequent causes of end-stage renal disease and are further exacerbated by maintenance dialysis. Diabetes and age > 40 years have been described as major risk factors for stroke occurrence after transplantation, and kidney recipients may also present post-transplant polycythemia with consequent hypercoagulability [40]. Moreover, mTOR inhibitors, CsA (and TAC to a lesser extent) may contribute to worsening dyslipidemia, thus increasing the risk of ischemic stroke [41, 42]. CNIs and steroids can also be responsible for hypertension (CsA more than TAC) and diabetes (TAC more than CsA) development or worsening, which represent leading causes of both ischemic and hemorrhagic cerebral events [43, 44]. In general, transplant patients suffer from an increased risk of ischemic rather than hemorrhagic events, but mortality is higher with the latter. The risk of aneurysm rupture and subarachnoid hemorrhage is increased in patients with a diagnosis of autosomal dominant polycystic kidney disease. Proper management of established risk factors is crucial in the follow-up of kidney transplant recipients, with routine carotid artery assessment by Doppler imaging playing a pivotal role.

#### Posterior reversible encephalopathy syndrome

Posterior Reversible Encephalopathy Syndrome (PRES) is a severe neurological complication caused by vasogenic edema, which can lead to permanent deficits when not properly identified and treated [45]. It usually affects the posterior cerebral circulation and the clinical presentation can include nausea, headache, visual impairment, seizures and consciousness alteration. Recently identified alternative presentations involve spinal cord PRES, subacute diencephalic angio-encephalopathy, as well as posterior fossa edema and hydrocephalus [45]. Common radiological lesions are localized to the parieto-occipital or posterior frontal cortical–subcortical regions, and are usually reversible [46]. Management is based on blood pressure control, supportive therapy and correction of electrolyte disorders, especially magnesium ones. Rapid withdrawal of the offending drug appears to prevent complications and hasten recovery [47]. Antiepileptic drugs should be used to treat seizures, keeping in mind that these drugs activate CYP450 and may reduce the bioavailability of glucocorticoids and CNIs. In particular, valproic acid, fosphenytoin and carbamazepine use may result in both enzymatic and protein displacement [48]. Safer alternatives with regard to their drug interaction potential may be represented by Levetiracetam, Gabapentin, Pregabalin and Lacosamide [49]. Anesthesia and mechanical ventilation should be instituted in generalized status epilepticus. Most frequently, immunosuppressant-related PRES occurs within the first year after transplantation (mostly in the first month), even though late-onset cases have been described [50]. CNIs are the immunosuppressive drugs most frequently associated with PRES development through brain endothelial and astrocyte damage [51]. CNIs have been shown to cause PRES even at therapeutic serum concentrations and without concomitant hypertension. However, cases of PRES related to rituximab and mTOR inhibitor treatment have also been reported [52, 53].

### Acute and subacute clinical presentations

#### Infections

Central nervous system infections are extremely frequent in renal transplant recipients, and represent 9–10% of all neurological complications in this population [54]. The subtle clinical presentation typically displayed by these patients can hinder prompt diagnosis and timely treatment, both of which are crucial to prevent a fatal evolution. Indeed, immunosuppressive drugs, due to their anti-inflammatory effects, can alter meningeal signs and clinical features typical of CNS infections. Patients may only report mild symptoms like headache, fever and confusion, which can abruptly evolve to focal neurological deficits and consciousness impairment. Thus, transplant recipients should be evaluated with a lower threshold for suspecting CNS infections compared to the immunocompetent population. In addition, CNS infections can be unusually severe in these patients, making prompt recognition and pathogen identification crucial.

To narrow down the exceptionally broad differential diagnosis of responsible infectious agents, time from transplantation should always be considered since specific pathogens are more likely to be responsible at different time points (Table 1). In the first month, nosocomial or pre-existing pathogens (e.g. latent tuberculosis reactivation) are most likely to be identified. During this time-frame, transplantation recipients may also suffer from donor-derived infections. The infectious risk is highest from one month to six months after transplantation, when doses of immunosuppressive drugs are higher and atypical pathogens are more prevalent. Fungal and viral opportunistic infections are more frequent: among these, cytomegalovirus (CMV) is the most common (up to 8% of renal graft recipients) and may cause meningeal and retinal complications. Many transplant centers recommend CMV prophylaxis with valacyclovir or ganciclovir in high-risk patients such as seronegative recipients who receive a graft from seropositive donors and/or patients treated with T cell-depleting antibodies. Other centers prefer to start preemptive treatment when signs of virus replication are found before CMV disease develops [55]. Fungal infections are less frequent, but they are burdened by a high mortality rate, with cryptococcus neoformans displaying the highest incidence in this category [56].

**Table 1:** Pathogens causing central nervous system infections according to time from renal transplantation

Time after transplant	Primary mechanism	Pathogen	Clinical presentation	Diagnosis	Treatment
First month (donor- and recipient-derived infections due to common nosocomial pathogens)^a^	Typical organisms	*Streptococcus pneumoniae, Neisseria meningitidis, Haemophilus influenza*	Acute meningitis (headache, fever, malaise, altered mental status, meningismus)	CSF analysis (frequently cloudy, neutrophilic predominance, ↓ glucose, ↑ protein)	Ceftriaxone 2 g i.v. q12h (for *S. pneumoniae* add vancomycin 15–20 mg/kg i.v. q12h and rifampicin 600 mg i.v./orally q12h if penicillin and cephalosporin resistant) Treat for at least 10 days (for *N. meningitidis,* treatment can stop for patient recovered by day 5)
		*Herpes Simplex Virus*	Acute meningoencephalitis typically involving the temporal lobe	CSF PCR, MRI/MRA	Acyclovir 10 mg/kg i.v. q8h for up to 21 days
	Opportunistic bacterial infection	*Listeria monocytogenes*	Acute meningitis, rhombencephalitis, abscesses, myelitis	CSF analysis (frequently limpid, neutrophilic predominance, ↓ glucose, ↑ protein)	Ampicillin/amoxicillin 2 g i.v. q4h or co-trimoxazole 10–20 mg/kg q6h. Treat for at least 21 days
	Recipient-derived infection	*Aspergillus fumigatus*	Brain abscesses, sinusitis, infarction, meningitis, aneurysms	CSF galactomannan, MRI/MRA, brain biopsy	Antifungals including voriconazole and amphotericin B
	Latent infection	*Mycobacterium tuberculosis*	Cerebral tuberculoma, meningitis, myelopathy	Screen with IFNγ-release assay. CSF analysis (↓ glucose, leukocytosis with lymphocytic predominance, ↑ protein) and CSF culture	Minimum of 9 months: isoniazid 5 mg/kg/day (+ pyridoxine 50 mg) + rifampicin 10 mg/kg day; Minimum of 2 months: pyrazinamide 25–30 mg/kg/day + streptomycin 15 mg/kg day or ethambutol 15–20 mg/kg/day
		*HHV-6*	Encephalitis, convulsions (mostly children)	Serum and CSF PCR	Ganciclovir, foscarnet, or cidofovir
1–6 months (early opportunistic infections)	Opportunistic bacterial infection	*Nocardia asteroides*	Meningitis, brain abscess	CSF culture (prolonged incubation)	Imipenem (500 mg i.v. q6 h) + Amikacin (10–15 mg/kg/d) or Trimethoprim/sulfamethoxazole (15 mg/kg day)
	Latent or opportunistic viral infection	*Cytomegalovirus*	Encephalitis, retinitis, myelitis	Ependymal enhancement on MRI, CSF PCR	Ganciclovir 5 mg/kg q12h i.v. or foscarnet 60 mg/kg q8h i.v
		*Varicella Zoster virus*	Skin lesions, multifocal vasculopathy, cranial nerve palsy, cerebellar ataxia, meningo-myelitis, post-herpetic neuralgia	CSF and serum PCR	Intravenous acyclovir 10 mg/kg q8h, foscarnet 90–120 mg/kg/day i.v., ganciclovir 5 mg/kg/day i.v.. Consider valacyclovir prophylaxis after infection is fully treated to prevent reactivation
		*Epstein-Barr virus*	Meningoencephalitis, aseptic meningitis, seizures, PTLD	CSF PCR, histopathology for PTLD	Immunosuppression reduction, rituximab and donor T-cell infusions for PTLD
> 6 months (late opportunistic infections)	Community-acquired	*Cryptococcus neoformans*	Meningitis, meningoencephalitis, cryptococcomas	CSF antigen testing (lateral flow assay), culture and PCR	Amphotericin B + 5-flucytosine. Lifelong treatment with fluconazole even after CSF cultures are negative
		*Candida species*	Sepsis, meningitis, abscesses	Pseudohyphae visualization, CSF PCR and culture	Fluconazole, voriconazole, amphotericin B
		*Toxoplasma gondii*	Meningoencephalitis, brain and spinal cord abscesses	Serum and CSF PCR, ring-enhancing lesions on imaging, biopsy of involved tissues (tachyzoites)	Pyrimethamine + sulfadiazine + leucovorin
	Latent or opportunistic viral infection	*JC virus*	Progressive multifocal leukoencephalopathy	CSF PCR	Consider reducing immunosuppression. Poor prognosis

*CSF* cerebrospinal fluid, *i.v.* intravenous, *MRI* magnetic resonance imaging, *MRA* magnetic resonance angiography, *PCR* polymerase chain reaction, *PTLD* post-transplant lymphoproliferative disease

^a^Arbovirus infections including West-Nile Fever and Chikungunya also need to be considered in patients and donors from geographical areas in which such diseases are endemic

Six months after transplantation, the infection risk starts to decrease and the most frequently encountered pathogens are EBV, Cryptococcus neoformans and JC virus, that can lead to PML, which is a fatal neurological condition characterized by subcortical white matter lesions with different neurological manifestations and without any effective treatment [57]. Nivolumab, a monoclonal antibody against PD-1 (programmed death-1), failed to obtain any improvement in transplant recipients [58], although stopping or reducing immunosuppressive drugs was successful in some cases [59]. Notably, infusion of allogeneic BK virus-specific T cells achieved alleviation of clinical signs and clearance/reduced load of JC virus in the cerebrospinal fluid (CSF) in two immunosuppressed patients [60].

The work-up for CNS infection is based on lumbar puncture, blood cultures and brain imaging. Cerebrospinal fluid analyses should include glucose and protein concentration, polymerase chain reactions for a comprehensive panel of viral and mycobacterial nucleic acids, cryptococcal antigen, cytology and microbiological cultures. Whenever available, contrast-enhanced MRI is the preferred radiological exam, since it can highlight leptomeningeal enhancement, parenchymal inflammation and potential abscesses.

It is important to rule out the cytokine-release syndrome as a differential diagnosis, since its manifestations can resemble those of CNS infection with vomiting, fever and seizures. This is a rare but severe systemic inflammatory response due to initial immune system activation after treatment with antilymphocyte antibodies (e.g. Rituximab, Alemtuzumab and ATG), used for induction or anti-rejection therapy [61]. These serious side effects are more frequent in patients older than 65 years, however premedication with methylprednisolone, paracetamol and antihistamines may reduce the frequency and severity of side effects.

Treatment of CNS infections includes empiric antimicrobial therapy based on ceftriaxone, ampicillin and acyclovir until the causal pathogen has been identified. A central issue with the use of antiviral drugs in kidney transplant recipients is represented by their potential nephrotoxicity. Cidofovir and, to a lesser extent, acyclovir may cause direct tubular toxicity and can also induce crystal deposition in the kidney that may result in renal failure. Hydration, slow intravenous administration and dose reduction in case of reduced glomerular filtration rate are important preventive measures. Azole antifungals are significantly metabolized by CYP3A4 and can therefore inhibit the catabolism of CsA, TAC and mTOR inhibitors, thus extreme caution is advised when using these agents in kidney transplant recipients.

Immunosuppressive drugs are usually tapered in the acute phase, unless the patient’s history suggests a disproportionally high risk of graft rejection. Indeed, infections pose a challenge in balancing antimicrobial therapy and inflammatory response in transplant recipients. A reduction of the immunosuppressive regimen to contain the infection might also cause immune reconstitution inflammatory syndrome [62]. This condition represents a diagnosis of exclusion and needs to be considered when patients present novel or worsening neurological clinical/radiological features associated with negative cultures.

#### Psychiatric disorders

Glucocorticoids are extensively employed as maintenance immunosuppression and for the treatment of acute rejection. Patients treated with glucocorticoids can suffer from different behavioral disorders, ranging from minor mood alterations and confusion to severe psychotic reactions [63]. Dose reduction may be sufficient to reverse or improve symptoms, even though antidepressant or neuroleptic treatment is required in selected cases. In case of psychosis or mania, prophylaxis with lithium and olanzapine may be considered. The risk of these side effects is increased by older age, previous history of psychiatric disorders, steroid dose, and treatment duration. Behavioral disorders can also complicate therapy with CNIs and mTOR inhibitors. The BENEFIT and BENEFIT-EXT trials compared patients undergoing maintenance immunosuppression with belatacept or CsA and observed that patients in the latter group displayed higher frequency of emotion-related side effects, such as depression, anxiety, or restlessness [64].

### Chronic clinical presentations

#### Malignancy

Transplant recipients have a well-known increased risk of malignancies compared to the general population, which is a direct consequence of reduced immuno-surveillance and increased susceptibility to infections with oncogenic viruses. The risk of developing systemic lymphoma in the first year after renal transplantation has been estimated to be 20 times higher than in the general population [65]. Almost 30% of all post-transplant lymphoproliferative disorders (PTLD) affect the CNS [66] and the most frequent malignant form is represented by large diffuse B-cell lymphoma, which accounts for only 2% of CNS malignancies in the general population. Many PTLD cases result from prior EBV infection, even though CSF and serum analyses might be negative for EBV-DNA and the specific pathogenic mechanism remains unclear [67]. EBV-negative patients are at high risk of cerebral lymphoma if the donor is a carrier of EBV. In such cases, immunosuppression should be maintained as low as possible and the use of belatacept should be avoided.

Clinical manifestations are variable depending on site and extension of the lesions with peripheral facial nerve palsy representing an early sign of PTLD with meningeal involvement. Definitive diagnosis usually requires a brain biopsy. Careful reduction of the immunosuppressive regimen based on the patient’s history of rejection is mandatory. Treatment options include rituximab, chemotherapy and surgery if the disease is localized.

#### Optic neuropathy

Optic neuropathy is a rare but well-recognized side effect of tacrolimus administration, which may occur from months to years after treatment initiation [68]. Patients may experience visual acuity impairment that can evolve into progressive severe visual loss, resulting in complete blindness. This complication usually affects both optic nerves, but unilateral symptoms have also been described, especially at disease onset [69]. Ophthalmic examination can show variable findings depending on disease stage, which may range from unremarkable to optic disc edema or pallor [69]. Brain and orbital MRI might reveal optic nerve inflammation in the form of contrast enhancement and T2 hyperintensity. The common clinical practice is to switch tacrolimus with another immunosuppressive agent, although unfortunately, visual impairment may be irreversible in many cases [68].

## Peripheral nervous system

### Acute clinical presentations

#### Guillain–Barré syndrome

Guillain–Barré syndrome (GBS) is the most common acute inflammatory polyradiculoneuropathy, affecting 0.8–1.9 patients per 100,000 per year [70]. The typical clinical evolution includes a first phase of ascending, mostly symmetrical, limb weakness and areflexia, progressing to its peak in 2–4 weeks, followed by a plateau phase that may last several months, and a subsequent recovery phase, in some cases incomplete with residual disability.

CMV is the most common viral cause of GBS, and it has been associated with most cases occurring in solid organ recipients, supporting the hypothesis that in these patients viral infections can act as relevant triggers for acute nerve inflammation [71]. GBS has been observed in numerous recipients of bone marrow transplant (BMT), particularly in those undergoing allogeneic BMT [72], but it is rarely seen after solid organ transplantation [73], although these patients are more susceptible to opportunistic infections as a result of immune suppression.

At present, 19 cases of GBS occurring in kidney recipients have been described in the literature [74, 75]. A systematic review already included the first 17 described cases, identifying an infectious trigger in 15 of them (88%), the most common being CMV infection or re-activation in seronegative patients receiving a kidney from a seropositive donor (80%); immunosuppressive treatment alone was possibly implicated in only two cases, although at least 13 patients (76%) were receiving CNIs [74]. Patients treated with standard GBS therapy based either on immunoglobulins (0.4 g/kg/day for 5 days) or plasma exchange (three cycles) achieved full neurological recovery. Immunosuppressive (TAC and/or MMF) dose reduction or suspension was reported in only three patients.

A recent single-center retrospective analysis identified 2 patients developing GBS in a cohort of 143 alemtuzumab treated kidney recipients, 92% of whom were receiving TAC-based immunosuppressive therapy (including the 2 GBS cases), within a mean interval of 6 months from alemtuzumab infusion. No recent infection was identified in either case [75]. However, the study found no causal link between monoclonal antibody therapy and the development of GBS, and therefore the role of possible reactivation of CMV, at least in one of the two patients who was seropositive at the time of transplantation, cannot be excluded as a predisposing factor for GBS.

Taken together, these data confirm that GBS is rare in renal transplant recipients, suggesting that immunosuppression gives a certain degree of protection. This hypothesis is supported by the immunomodulatory effects of MMF, which could mitigate the immune mechanisms underlying GBS development, and the effective use of CsA in the treatment of inflammatory neuropathies [76]. However, more data are needed to understand the correct management of immunomodulating treatment in these patients.

An infectious etiology, particularly CMV re-activation, is postulated or strongly suspected in most of the cases described, which represents a higher percentage than that reported for the general adult population. Moreover, all patients receiving at least one approved immunotherapy for GBS achieved full recovery, except for one CMV-associated subject who did not fully recover lower limb strength.

Therefore, the combination of antiviral medications with standard GBS therapy seems to determine the best outcome and should be offered to all patients in whom a viral etiology cannot be excluded. More information is needed to understand which is the best therapeutic approach.

#### Myasthenia gravis

Myasthenia gravis (MG) is the most frequent disorder of the neuromuscular junction. It is associated with antibodies directed against the postsynaptic membrane at the muscle endplate, manifesting as fatigue and muscle weakness. An abrupt worsening of symptoms, called myasthenic crisis, may lead the patient to require respiratory support. It can occur spontaneously as part of the natural history of the disease itself, but it can also be triggered by a number of factors; infections, pregnancy, childbirth, surgery and different drugs.

To date, the literature reports only anecdotal cases of myasthenic crises in patients treated with CNIs following renal transplantation [77, 78]. However, it was not possible to establish a direct correlation with immunosuppressive anti-rejection therapy in any of these cases due to simultaneous confounding factors (i.e. surgical procedures, infection, antibiotic therapy). Moreover, glucocorticoids, azathioprine and CNIs are listed among the conventional immunosuppressants in the treatment of MG [79]. In the most recently described case, muscle weakness began one year after the transplant and the authors also excluded any correlation between myasthenic crisis and alemtuzumab, which was used as induction therapy 1 year before the onset of neuromuscular symptoms [78].

A direct association between MG and renal transplant is therefore unlikely, but patients with an established MG diagnosis who undergo kidney transplantation must be tightly monitored. It is crucial for physicians to be aware that MG symptoms are likely to deteriorate suddenly during the perioperative period. Although there are few supporting data in the literature, conventional myasthenic crisis treatments (pyridostigmine, i.v.immunoglobulins, plasma exchange) also seem to be effective in these patients.

### Chronic clinical presentations

#### Tremors

Tremors affect approximately one-third of renal transplant recipients, with variable degrees of symptom severity ranging from mild to highly incapacitating [80]. CNIs are mostly responsible for this disorder, possibly due to their serotonin-depletion effect on neurons [81]. Affected patients usually develop fine resting and action tremors involving the upper extremities, which may significantly impact on daily activities. Although tremors may develop following exposure to any CNI, TAC has been more frequently associated with this disorder compared to CsA [82–84].

CNI-induced tremors are generally regarded as a dose-dependent effect due to over-exposure. Monitoring TAC and CsA trough levels is frequently used as a method to assess CNI exposure, but blood levels do not always correlate with intracellular concentrations and pharmacologic effect [85]. CNI-induced tremors may also develop in patients with TAC/CsA blood levels in the “correct” therapeutic range.

Dose reduction of CNIs may lead to significant improvement in symptoms, and some patients may also respond to beta-blockers. Extended-release formulations of tacrolimus may provide some benefit over conventional forms, but randomized controlled trials in this context are still lacking [86, 87].

#### Hearing complications

Hearing loss, tinnitus or otalgia may develop in CNI-treated patients. A questionnaire administered to 521 liver transplant recipients under treatment with TAC reported that 14% developed hearing loss [88]. Hearing loss may also occur in pediatric renal transplant recipients treated with high doses of TAC [89] or CsA [90]**.** It has been suggested that deafness might be initiated by a sudden spike in the TAC serum level, which is later worsened by its cumulative toxic effect [91], accordingly, dose correction may lead to hearing recovery.

#### Toxic neuropathies

Peripheral toxic neuropathy is the most frequent neurological adverse event in renal transplant patients, with an overall prevalence of more than 2% [54]. Among drugs used for immunosuppression in kidney transplant patients, CNIs are responsible for most of the toxic effects on the peripheral nerve. A painful sensory peripheral neuropathy is also very frequent in patients treated with bortezomib, a proteasome inhibitor used for antibody-mediated rejection.

Patients receiving CNI therapy show nerve function abnormalities, quantified through nerve excitability measures, consistent with nerve membrane depolarization. In 2007, the first two kidney recipients who developed sensorimotor neuropathy while on therapy with TAC were reported, showing different electrophysiological findings (either predominantly demyelinating or axonal) of neuropathic damage [92]. One of these patients showed clinical, CSF and neurophysiologic findings suggestive of relapsing chronic inflammatory demyelinating polyneuropathy (CIDP), which was responsive to repeated i.v. immunoglobulin treatments. This case has many similarities to that of a kidney transplant recipient who developed life-threatening sensory-motor peripheral neuropathy 10 years after the graft, suggesting a diagnosis of CIDP [93]. The authors postulated a causal role of TAC, hinted at by the temporal relationship, the long-term response after cessation of treatment and the exclusion of other causes, and they theorized a TAC-induced trigger acting on an inflammatory substrate given the quick response to v i.v. immunoglobulin treatment. TAC was switched to sirolimus, which was well tolerated. A prospective study on solid organ recipients reported three more patients developing CIDP within a time interval between 8 and 36 months while on CsA 400–600 mg/day [94]. All these patients received a final diagnosis of typical definite CIDP. They continued to take CsA and showed brilliant response to i.v. immunoglobulin treatment and a monophasic disease course with no relapses observed within a follow-up period of at least 4 years.

Several factors may contribute to the occurrence of CIDP in renal transplant patients. CNIs still play an unclear role since they have been described as provoking demyelinating neuropathy but also as having positive effects on CIDP [95]. Patients receiving CNIs should be carefully monitored for neuropathic symptoms, and CIDP should be suspected in all patients with compatible clinical and neurophysiological characteristics, after excluding other etiologies. Reducing CNI dose or switching to alternative drugs such as mTOR inhibitors should be considered in such patients.

Bortezomib-induced peripheral neuropathy is a well-known adverse event of the drug. A prospective study evaluated the toxicity profile of bortezomib in 51 kidney recipients undergoing antibody-mediated rejection [96]. The study revealed that 26.4% of patients showed typical sensory new onset or worsening peripheral neuropathy of mild degree. Patients exhibited improvement of symptoms at the end of treatment, with complete resolution of new onset peripheral neuropathy being observed in 91.7% of patients. Since bortezomib toxicity on peripheral nerves seems to be mild and reversible, patients should undergo periodic clinical assessments, with appropriate dose changes until suspension. Tricyclic antidepressants, selective serotonin-norepinephrine reuptake inhibitors, pregabalin and gabapentin should be used in the management of symptoms [97].

Finally, toxic neuropathy should be differentiated from CNI-induced pain syndrome, a rare but severe side effect. The disease should be suspected in patients complaining of movement-related, lower limb symmetric deep aching pain, especially soon after transplantation, and the diagnosis is confirmed by bone scintigraphy and magnetic resonance imaging. However, this syndrome is uncommon among kidney transplant patients, with a prevalence of 0.8–5.8% [98, 99]. Pregabalin may be administered as the drug of choice.

#### Autonomic neuropathy

The involvement of the autonomous system is common in patients with chronic kidney disease, especially due to the high prevalence of diabetes in this population. Autonomic neuropathies in these patients are frequently axonal, hence longer nerves (e.g. the vagus nerve) are affected earlier. Typical manifestations range from blood pressure disorders (orthostatic hypotension, increased peripheral vascular resistance and reductions in day-to-night blood pressure variations) due to impaired baroreceptor function, gastrointestinal symptoms (gastroparesis and alternating constipation/diarrhea) and genitourinary disorders (incontinence, erectile dysfunction). Some improvement in cardiovascular autonomic function has been reported in dialysis patients after renal transplantation [100], but other studies have described the persistence of autonomic dysfunction in renal transplant recipients [101, 102]. These effects may be in part due to the use of immunosuppressive drugs, which may affect autonomic function [103]. Symptomatic treatment may provide some relief, but no study to date has explored the effect of different immunosuppressive regimens on autonomic neuropathy.

#### Myopathy

Steroid-induced myopathy can arise after a variable period of time and involves proximal muscle weakness with atrophy [104]. These forms preferentially affect the lower extremities and patients sometimes experience myalgia or cramps. The diagnosis is clinical, muscular enzymes are usually not elevated and treatment is focused on steroid tapering.

## Prevention and management

Many neurological complications in transplant patients are related to comorbidity, hence early treatment of hypertension, diabetes mellitus and dyslipidemia is of paramount importance. Nevertheless, the best measure to prevent complications in an immunocompromised patient is to minimize immunosuppressive therapy. However, since this approach can expose transplant recipients to rejection and graft failure, knowledge of the immune status of a patient would be of great importance to decide the intensity of the anti-rejection therapy.

Several studies have been conducted in the search for tests able to assess the immunological status of transplant recipients, with the ultimate goal of predicting the risk of both rejection and of complications due to over-immunosuppression [105]. Monitoring donor-specific anti-HLA antibodies can be useful to predict the risk of antibody-mediated rejection and poor renal outcomes [106], but this method cannot provide quantifiable information regarding the degree of immunosuppression. Assays that test recipient T-cell reactivity against donor cells (mixed lymphocyte reaction and cell-mediated lympholysis) have been advocated as a possible tool to gauge immunosuppression intensity [107], but these techniques are time-consuming and difficult to standardize. Enzyme-linked immunosorbent spot (ELISPOT) assays have been used to assess cytokine production from donor-specific T-cells, to investigate viral infection susceptibility, and to attempt withdrawal of immunosuppressive medications in tolerant patients. However, most studies conducted so far have been retrospective, and conflicting results have been reported [108], thus further studies are needed to validate the role of this assay as a marker of the immune status of transplant recipients [105].

Two additional assays were developed to characterize immunosuppression intensity, based either on the quantification of adenosine triphosphate released by CD4+ T cells [109], or on the measurement of IFNγ levels in blood samples after exogenous stimulation of both the innate and adaptive immune systems [110]. Despite initial enthusiasm, none of these assays have been translated to clinical practice.

More recently, monitoring replication of torque teno virus (TTV), a non-enveloped single-strand DNA virus that is part of the human virome, has been proposed as an additional tool to assess immune function in renal transplant recipients. In a prospective observational study, low TTV loads during the first year post-transplant were associated with rejection, while high levels predicted infectious complications [111]. An optimal TTV range was defined, but further studies will be required to evaluate whether TTV-guided immunosuppression could improve short- and long-term outcomes in these patients.

Since standard methods to assess the intensity of immunosuppression are still lacking, physicians should try to adjust immunosuppressive therapy on the basis of features such as age, clinical history, and comorbidities. Elderly patients experience a decline in natural and adaptive immunity [112], while the activity of cytochrome P450 enzymes is reduced with advanced age [113]. Since these enzymes regulate the catabolism of CNIs, mTOR inhibitors and glucocorticoids, accumulation of these drugs can occur in elderly individuals. In addition, hypertension, diabetes, and cardiovascular and cerebrovascular disease are common in older candidates to kidney transplantation. Therefore, in older individuals, immunosuppressive therapy should be tailored to a less vigorous approach by avoiding or withdrawing glucocorticoids after the induction phase [114].

## Conclusions

Kidney transplant recipients are at risk of various neurological complications, mainly due to immunosuppressive drugs, and in general to their pre- and post-transplant comorbidities. These side effects can range from tremor, dizziness and paresthesia to more complex and life-threatening clinical conditions. Prevention, early diagnosis and appropriate management of these complications are critical to prevent irreversible lesions. In the past, neurological adverse events mainly occurred within a few weeks from transplant and were frequently related to opportunistic infections. In more recent years, since CNIs and other effective immunosuppressive drugs have been made available, there have been tremendous advances in the field of organ transplantation with considerable improvement in the short- and long-term survival of the patient and of the kidney allograft. Since the main focus of this review is to provide a working summary of the more common and potentially severe immunosuppression-related neurological complications in a framework based on urgency and anatomic site, our work cannot be regarded as exhaustively comprehensive, and readers interested in any of the covered topics are encouraged to pursue additional information through the cited references. More data from basic and clinical studies are needed to address the knowledge gaps in both the pathogenesis and treatment of many of these complications, and clinical decisions should take into account inter-patient variability. For these reasons, we also encourage close collaboration between nephrologists and neurologists, not only to allow correct and rapid identification of these neurological disorders, but also to provide the best therapeutic strategy in managing the delicate balance between immunosuppression and serious adverse effects.
